# Immunological Evaluation for Personalized Interventions in Children with Tuberculosis: Should It Be Routinely Performed?

**DOI:** 10.1155/2020/8235149

**Published:** 2020-09-14

**Authors:** Laura E. Carreto-Binaghi, Esmeralda Juárez, Silvia Guzmán-Beltrán, María Teresa Herrera, Martha Torres, Alejandro Alejandre, José Arturo Martínez-Orozco, Eduardo Becerril-Vargas, Yolanda Gonzalez

**Affiliations:** ^1^Microbiology Research Department, Instituto Nacional de Enfermedades Respiratorias Ismael Cosío Villegas, CDMX 14080, Mexico; ^2^Pediatric Pulmonology Clinical Ward, Instituto Nacional de Enfermedades Respiratorias Ismael Cosío Villegas, CDMX 14080, Mexico; ^3^Clinical Microbiology Department, Instituto Nacional de Enfermedades Respiratorias Ismael Cosío Villegas, CDMX 14080, Mexico

## Abstract

Childhood tuberculosis (TB) is a significant public health problem and the ninth leading cause of death worldwide. Progression of *Mycobacterium tuberculosis* infection to active disease depends on mycobacterial virulence, environmental diversity, and host susceptibility and immune response. In children, malnutrition and immaturity of the immune system contribute to an inadequate immune response. Coinfections, though rarely described in TB, might be associated with host immune deficiencies. Here, we describe the immunological evaluation of eight pediatric patients infected with a member of the *M. tuberculosis* complex, most of them with concomitant pulmonary infections (bacteria, viruses, or fungi). We assessed the functionality of several innate immunity receptors, IL-12 receptor, and IFN-*γ* receptor, as well as the antioxidant levels (glutathione), which are essential mechanisms for fighting intracellular pathogens such as *M*. *tuberculosis*. This study is aimed at developing a thorough immunological evaluation of patients with TB and a coinfection.

## 1. Introduction

Tuberculosis (TB) is a leading infectious disease in adults, and children within the household contact are at a high risk of developing TB. One million cases of childhood TB are reported every year worldwide, and TB is a significant cause of morbidity and mortality in children from countries with high prevalence, particularly from Southeast Asia, Africa, and Western Pacific regions [1]. Children are more susceptible to developing the disseminated form of the disease; thus, there is a high rate of extrapulmonary TB. Also, the Human Immunodeficiency Virus (HIV) coinfection in childhood TB is highly prevalent [1], suggesting an impaired immune response associated with age.

Diagnosis of TB in children is difficult, considering that they develop a paucibacillary infection that might not be detected with the conventional laboratory techniques: Ziehl-Neelsen staining, culture, or polymerase chain reaction for *Mycobacterium tuberculosis* [2]. The diagnostic complexity is higher in cases of extrapulmonary TB [3]. Moreover, current immunological tests, such as the tuberculin skin test and the interferon-*γ* (IFN-*γ*) release assays, usually standardized for adult patients, present different responses in children, probably related to the age of disease presentation and their recent BCG vaccination status [4], and it is essential to consider that the diagnosis based in immunological responses also could be affected by the impaired immune response of the patient.

An effective immune response to mycobacterial infection requires both innate and adaptive immune responses, which is particularly important for the development of active or latent disease [5]. Innate responses mediated by Toll-like receptor (TLR)2, TLR4, and TLR9 and NOD-like receptors (NLR) NOD1 and NOD2 play a vital role in the induction of proinflammatory cytokines and antimicrobial responses following pathogen recognition [6–8]. After engulfing mycobacteria, mononuclear phagocytes produce interleukin 12 (IL-12), which stimulates IFN-*γ* production by T cells mainly through the IL-12 receptor (IL-12R). IFN-*γ*, in turn, binds to its receptor, further upregulating IFN-*γ*-responsive gene transcription. This gene transcription enables macrophage activation, differentiation, and production of tumor necrosis factor-*α* (TNF-*α*), which helps to control intracellular mycobacterial infection, and the formation and maintenance of a granuloma [9–11]. In addition, other molecules such as glutathione (GSH), which participate in the oxidative balance by scavenging oxidant radicals, enhance NK cell cytolytic activity and exhibit an inhibitory effect of the intracellular growth of *M. tuberculosis* [12]. The functionality of these mechanisms is necessary for infection clearance, and impairment of any of them may associate with susceptibility to TB.

The gene susceptibility to childhood TB relates to several immune dysfunctions [13–16]. Some examples include Mendelian Susceptibility to Mycobacterial Diseases (MSMD), which comprises different genetic disorders related to an impaired IL-12/IFN-*γ* axis response [17]; the simultaneous alteration of NOD2 and IFN-*γ* receptor (IFNGR) [18]; and polymorphisms of different genes that play essential roles in the induction of antimycobacterial immune responses, such as TLRs and vitamin D and cytokine receptors [19]. Additionally, inadequate concentrations of 25-hydroxyvitamin D are associated with respiratory tract infections in infants and children [20] and low levels of GSH with a decreased control of *M. tuberculosis* infection in individuals with HIV [21, 22] and individuals with type 2 diabetes [23]. Despite their relevance in the control of infection, immune responses are not routinely evaluated in children with TB, probably because of the limitation of biological samples suitable for immunological studies and the lack of guidelines to help in the decision-making.

**Figure 1:**
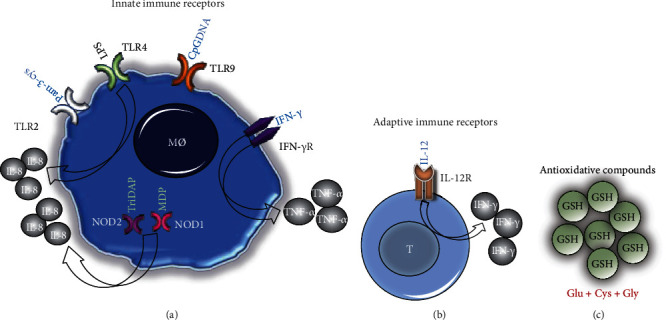
Strategy to assess the functionality of innate and adaptive immunity. (a) Innate receptors TLR2, TLR4, TLR9, NOD1, and NOD2 were stimulated with their synthetic ligands for the production of IL-8. IFN-*γ* receptor was stimulated with rhIFN-*γ* for the production of TNF-*α*. (b) IL-12 receptor was stimulated with rhIL-12 for the production of IFN-*γ*. (c) The cellular risk for oxidative damage was determined by measuring the extracellular reduced glutathione (GSH) in plasma.

This study is aimed at performing a feasible strategy to analyze multiple sets of immunological receptors using a small amount of blood from children with TB, in whom the available sample amounts may be a limitation to explore immunological mechanisms. In this report, we suggest an evaluation of immune responses to help the physicians to manage the complementary therapy according to immune response impaired. We used a cytokine production assay in diluted whole blood following stimulation with synthetic ligands of NLRs, TLRs, and IL-12/IFN-*γ* receptors, together with GSH evaluation, as a readout of the functionality of innate and adaptive immune responses in children with TB (Figure 1). Our data revealed that most of the patients had reduced responses to synthetic ligands of innate receptors. However, we did not identify a unique response pattern, which suggests that the impaired immunological mechanism is different in each patient; these data support the current concept of personalized medicine [24].

## 2. Materials and Methods

### 2.1. Pediatric TB Patients

We included eight pediatric patients with TB hospitalized at the National Institute for Respiratory Diseases Ismael Cosío Villegas (INER) in Mexico City. TB was diagnosed either by clinical or by radiological features after intradomiciliary TB contact or a positive TB test in clinical samples (*M. tuberculosis* culture, Ziehl-Nielsen staining, or nucleic acid amplification test). Concomitant infections were diagnosed by standard culture (bacteria or fungi), multiplex polymerase chain reaction (viruses), or specific polysaccharide detection (fungi). Most patients received treatment with isoniazid, rifampin, pyrazinamide, and ethambutol; however, treatment duration varied according to the clinical presentation and individual follow-up evaluation. As a reference, we also evaluated 16 healthy volunteers, with a mean age of 28 years (range 4-46 years); 55% of these volunteers were female.

### 2.2. Ethics Statement and Consent to Participate

This study was performed following the recommendations of the Committee of Ethics in the Research of INER. All patients, or their parents, and healthy volunteers gave written informed consent for this study, under the Declaration of Helsinki. The Institutional Review Board of INER approved this study (project number C02-18).

### 2.3. Blood Samples

We obtained 3 ml of heparinized venous whole blood by standard phlebotomy. Two milliliters of the whole blood was used for functional receptor evaluation. One milliliter was centrifuged, and the plasma samples were recovered for glutathione determination.

### 2.4. Innate NLR and TLR Receptor Evaluation

Innate receptors are essential to sense pathogens or their components and to induce an immunological response. The whole blood was diluted (1 : 4) in RPMI 1640 culture medium (Lonza, Walkersville, MD) supplemented with 10% of heat-inactivated human serum and plated in 96-well culture plates (Corning Costar Co., Corning, NY). The diluted whole blood was stimulated with NLR ligands: NOD1-TriDAP (10 *μ*g/ml) and NOD2-MDP (10 *μ*g/ml), and TLR ligands: TLR4-LPS (100 ng/ml), TLR2-Pam-3-cys (20 ng/ml), and TLR9-CpGDNA (5 *μ*g/ml), and incubated for 24 h at 37°C and 5% CO_2_ atmosphere. The supernatants were harvested and stored at -20°C until the evaluation of IL-8 production by ELISA, according to the manufacturer's instructions of the reagent kit (Probiotek, Monterrey, MX). Data are reported as pg/ml. There are no standard reference values for IL-8 plasma concentrations produced in the culture conditions here assayed in children. Thus, for this study, a low or high response was defined as values below or above 99% of the confidence interval (CI) of the healthy volunteer values, respectively. An adequate response was defined as the values within 99% CI of the healthy volunteer values.

### 2.5. Adaptive IL-12 and IFN-*γ* Receptor Evaluation

Cytokines from the axis IFN-*γ*/IL-12/IL-23, which play an important role in the intracellular growth control of pathogens, were studied. For the evaluation of IL-12R, 300 *μ*l of the whole blood diluted 1 : 1 in RPMI 1640 was stimulated with PHA (5 *μ*g), rhIL-12 (20 ng), and PHA (5 *μ*g) plus rhIL-12 (20 ng) and incubated during 48 h at 37°C and 5% CO_2_ atmosphere, and the culture supernatants were harvested and stored at -20°C until IFN-*γ* determination by ELISA [25, 26]. The low or high response was defined as IFN-*γ* values below or above the 99% CI of the healthy volunteer values, respectively. For the IL-12R, increases in the IFN-*γ* production after rhIL-12 plus PHA stimulation, relative to PHA alone, are expected, while for IFNGR, 300 *μ*l of the whole blood diluted 1 : 1 in RPMI 1640 were stimulated with LPS (100 ng) and LPS (100 ng) plus different rhIFN-*γ* concentrations (100, 500, and 1000 IU) and incubated during 24 h, and the culture supernatants were harvested and stored at -20°C until TNF-*α* determination by ELISA [25, 26]. Results were reported as pg/ml. The low or high response of IFNGR was defined as a TNF-*α* production outside the 99% CI of the healthy volunteer values. An increase of TNF-*α* production for LPS+rhIFN-*γ* vs. LPS alone is expected.

### 2.6. The Antioxidant Tripeptide GSH Quantitation

GSH levels were measured in plasma using the GSH reductase enzyme method previously described [27]. For this test, GSH reacts with 5,5′-dithio-bis (2 nitrobenzoic acid) (DNTB) to form 5-thio-2-nitrobenzoic acid (TNB), detectable in the spectrophotometer at 412 nm. The test is specific for GSH based on the specificity of the GSH reductase. The accumulation rate of TNB is proportional to the concentration of GSH in the sample. Freshly prepared DTNB and GSH reductase solutions were added to each plasma. Followed by the addition of *β*-NADPH. The absorbance was measured immediately and at 30 s intervals for 2 min. The rate of change in absorbance was compared to that of GSH standards. Data were expressed as plasma-GSH (nmol/mg protein). The glutathione concentration of each patient was compared with the median GSH concentration from healthy volunteers. A low response was defined as values below the 99% CI of GSH values in healthy volunteers.

## 3. Results

### 3.1. Clinical and Demographic Data of Pediatric Patients

We evaluated eight patients with tuberculosis, and their clinical and demographic data appear in Table 1. Most of the patients (87.5%) were female, and their ages varied from 1 to 16 years (mean age was eight years). The clinical presentations of these patients were pulmonary (five patients), lymph node (two patients), or bone TB (one patient). The patients with pulmonary TB were selected with coinfection with fungi, viruses, or other bacteria; the patients with extrapulmonary TB were selected because they likely had an alteration in their immune responses. Pulmonary TB patients received treatment for 6 or 18 months, together with the complementary treatment for the concomitant infection. Extrapulmonary TB patients were treated for 18 or 24 months.

### 3.2. The Innate (NLR/TLR Receptors) Immune Responses of Pediatric Patients

**Figure 2:**
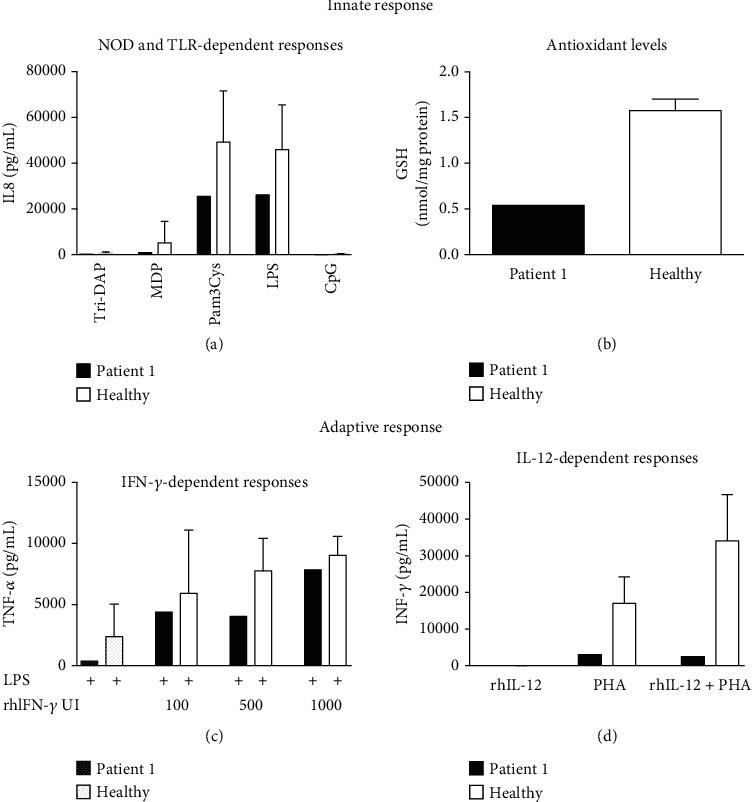
Graphical expression of the results of the immunological evaluation. As an aid in the interpretation of results, the graphical comparison of innate (a, b) and adaptive (c, d) responses between patient one and the mean of the control group is depicted.

Parameters of the innate immunity are candidate biomarkers for TB susceptibility, diagnosis, and treatment outcome in pediatric patients [28]. We evaluated the production of IL-8 in response to specific NOD1, NOD2, TLR2, TLR4, or TLR9 ligands in children with TB. The response of NLRs and TLRs of each patient appears in Table 2. We observed a low response to innate NLRs in P1 and P4. Contrariwise, we observed a high response to NLR and TLR ligand stimulation in P7. The P2, P3, P5, P6, and P8 showed an adequate response to NLR and TLR ligands (Table 2, Figure 2 and supplementary figure 1).

### 3.3. The GSH Levels as a Marker for Oxidative Stress in Pediatric Patients

Oxidative stress has been considered a significant contributor to the development and prognosis of TB [29]. GSH's ability to scavenge oxidant radicals contributes to diminishing oxidative stress. We evaluated the level of antioxidant GSH and observed adequate values (equivalent to healthy volunteers) in P2-P8, whereas low levels of glutathione were observed in P1, suggesting an enhanced cellular oxidation state (Table 2, Figure 2, and supplementary figure 1).

### 3.4. The Adaptive (IFN-*γ* and IL-12 Receptors) Immune Response in Pediatric Patients

The immunologic function of IL-12 and IFN-*γ* receptors has been studied in blood cells using methods described elsewhere [30, 31]. A defect in the IL-12R gene in a patient results in a nonincreased IFN-*γ* production in response to PHA plus rhIL-12 stimulation [32]. We observed that P1 did not increase the IFN-*γ* production after PHA plus rhIL-12 stimulation, suggesting a probable genetic deficiency of IL-12R. Additionally, we observed a low IFN-*γ* production after PHA plus rhIL-12 stimulation in P3, P4, P6, and P7, suggesting immunosuppression in these patients. We also observed a high production of IFN-*γ* in P2, suggesting an exacerbated immune response (Table 2 and supplementary figure 1).

When evaluating the IFNGR functionality, the lowest TNF-*α* production in response to LPS plus IFN-*γ* stimulation has been associated with a defect in the IFNGR gene [32]. We observed a low response to high concentrations of IFN-*γ* stimulation plus PHA stimulation in P7 and P8, suggesting a probable defect in the IFNGR (Table 2 and supplementary figure 1).

## 4. Discussion

Childhood tuberculosis, particularly extrapulmonary clinical forms and pulmonary forms associated with coinfections, might reflect an impairment of host defenses or a major immune defect. The assessment of the immune system's quantitative and functional efficacy is necessary to identify the underlying host factors involved in these forms of the disease. There is no standard for immunological evaluation in children; however, in this study, we developed a strategy to evaluate innate and adaptive immune response receptor functionality in children with TB using a minimum amount of whole blood and synthetic stimulants.

Using as little as 3 ml of whole blood, we performed three sets of immunological evaluations. First, we evaluated the innate responses by stimulating TLR2, TLR4, TLR9, NOD1, and NOD2 receptors in terms of IL-8 production. This evaluation revealed that most patients had adequate responses, and two patients exhibited low NOD1- and NOD2-dependent responses. An insufficient innate proinflammatory cytokine production has been associated with the inability to clear infections early, which suggests an underlying immunological susceptibility for those two patients [33, 34]. One patient exhibited exacerbated proinflammatory responses. This patient is at higher risk of complications because the innate immune system is the first line of defense against infections and is also responsible for a widespread inflammation that might cause tissue and organ injury.

Second, we determined the glutathione (GSH) concentration in plasma. The antioxidant GSH protects all cells against oxidizing agents, free radicals, and reactive oxygen intermediates and has both antimycobacterial effects and immune-modulating properties. Thus, GSH deficiency or its imbalance causes cellular risk for oxidative damage in pathological conditions such as TB [35, 36]. We observed reduced GSH values in one patient suggesting a deficiency in innate immunity that makes it more susceptible to oxidative stress. It may also be of relevance to determine adverse drug effects.

Third, we explored the IFN-*γ*/IL-12 axis-dependent responses, where genetic deficiencies in this pathway are referred to as MSMD [37]. We found partial IFN-*γ* receptor responses in two patients suggesting the need for further genetic assessments to identify a probable genetic mutation. Additionally, we observed a low response of the IL-12 receptor in four patients and inadequate response in one patient, suggesting an immunosuppressive state associated with susceptibility to TB infection.

The present evaluation of immune responses was designed to help the clinicians make therapeutic decisions. All elements in this response are crucial for fighting mycobacterial infections and can be measured at the immunology laboratory by stimulation with synthetic ligands using low amounts of whole blood. Also, this evaluation is rapid and relatively inexpensive. The study of the immunity status in children with TB may be useful to address several issues of childhood TB. For example, it could help in decision-making for immunomodulatory therapy, which may focus on inhibiting the inflammatory response for exacerbated innate responses, using immunostimulants where blocking inflammation may be detrimental, or administering antioxidants to prevent cellular damage when needed. It could also help determine whether further genetic evaluations are required. Other potential uses for this strategy are biomarker discovery in young children, evaluation of treatment shortening trials, and the evaluation of novel regimes. The latter would be particularly useful for TB caused by *M. bovis*, considering that treatment duration is usually long because of the exclusion of pyrazinamide since all strains of *M. bovis* are resistant to it [38]. The potential uses are vast and favor the notion of using immunological screening at the routine laboratory in childhood tuberculosis clinic.

Our strategy has limitations, however. We included adults as controls because healthy children are challenging to evaluate for ethical reasons. However, innate responses to synthetic ligands in children of 1, 2, and 5 years old are similar to that of the adults, and PHA-dependent IFN-*γ* production by PBMC of children aged 5 or older is similar to that of adults [39]. Our control group consisted of a mestizo Mexican population; reference values should be obtained for local populations with specific genetic and nutritional backgrounds. An additional limitation of this study is that we are unable to discriminate specific cellular impairments. The evaluation of soluble components does not provide information on the cellular source, and further experiments will be required to determine whether T cells, NK cells, neutrophils, or macrophages are compromised [40]. However, more complex assays may follow the initial screening if warranted.

## 5. Conclusion

Immunological evaluations are not routinely performed in children, but its utility urges to implement a strategy that may also aid in other relevant issues, such as the selection of optimal therapy, prognostic assessment, immunotherapy interventions, and genetic counseling, as well as to identify individual protective factors. This work is a proof of concept study that needs to be confirmed with a larger group of patients and correlated with the outcome. Our strategy purports affordable tests for widespread use with potential for use in tailored host-directed therapies.

## Figures and Tables

**Table 1:** Clinical and demographical data of TB patients.

Subjects	Age	Sex	BMI (kg/m^2^)	TB type	Concomitant infections	TB treatment	Treatment duration
Patient 1	14	F	15.2	Pulmonary	Unknown fungus	HRZE	18 months
Patient 2	5	M	13.6	Pulmonary	*M. catarrhalis*	HRZE	6 months
Patient 3	12	F	19.3	Lymph node	No	HRZE	2 years
Patient 4	16	F	18.5	Bone	No	HRZE	2 years
Patient 5	13	F	20.3	Lymph node (RR)	No	H, Lfx, Dlm, Lzd, Cfz	18 months
Patient 6	1	F	14.7	Pulmonary	Rhinovirus	HRZE	6 months
Patient 7	1	F	11.5	Pulmonary	Flu A H1N1	HRZE	6 months
					*Cladophialophora* spp.		
Patient 8	4	F	13.2	Pulmonary	*Penicillium* spp.	HRZE	6 months

TB: tuberculosis; RR: rifampin resistant; H: isoniazid; R: rifampin; Z: pyrazinamide; E: ethambutol; Lfx: levofloxacin; Dlm: delamanid; Lzd: linezolid; Cfz: clofazimine.

**Table 2:** Innate (NLR/TLR and IFN-*γ* receptors) responses, antioxidant levels, and adaptive IL-12 receptor responses elicited within the study group.

Stimulated receptor	NOD1^a^	NOD2^a^	TLR2^a^	TLR4^a^	TLR9^a^		Antioxidant levels^b^		IFN-*γ* receptor^c^					IL-12 receptor^d^			
Subject	TriDAP	MDP	Pam-3-cys	LPS	CpG	Type of response	GSH (nmol/mg protein)	Type of response	LPS	LPS+rhIFN-*γ* 100 IU	LPS+rhIFN-*γ* 500 IU	LPS+rhIFN-*γ* 1000 IU	Type of response	rhIL-12	PHA	rhIL-12+PHA	Type of response
Patient 1	296	990	25480	26090	126	Low NLR	0.5	Low	364	4391	4041	7841	Adequate	11	2461	3029	Low
Patient 2	891	2393	24672	55372	208	Adequate	1.6	Adequate	nd	nd	nd	nd	nd	0	23793	65315	High
Patient 3	1142	10393	48171	31838	0	Adequate	1.2	Adequate	2071	3989	4013	2460	Adequate	0	469	12597	Low
Patient 4	0	1153	28253	24187	0	Low NLR	1.7	Adequate	845	3181	9738	8647	Adequate	0	4619	15450	Low
Patient 5	2109	10293	54982	45682	382	Adequate	2.1	Adequate	2146	2744	1618	3228	Adequate	10	13172	25972	Adequate
Patient 6	0	32166	59726	38926	0	Adequate	1.3	Adequate	1905	567	1861	1099	Adequate	2	7754	20511	Low
Patient 7	21680	30780	114900	52480	1690	High	1.6	Adequate	144	187	206	285	Low	5	248	1677	Low
Patient 8	0	2917	90728	72478	0	Adequate	1.4	Adequate	1826	348	0.2	0	Low	nd	nd	nd	
Healthy donors (*n* = 16) median, 99% CI [LL, UL]	803 [160.7, 1967]	5158 [1210, 23050]	49189 [23306, 74664]	45903 [25933, 92485]	163.3 [0.0, 789.2]	Reference value	1.575 [1.160, 1.760]	Reference value	2365 [197.3, 7403]	5924 [861.5, 12150]	7754 [529.9, 10925]	9017 [844.9, 11557]	Reference value	17.37 [0.0, 78.34]	17036 [9337, 25752]	34088 [27614, 58018]	Reference value

^a^Readout IL-8 production, pg/ml. NLR and TLR response was defined as low or high: outside LL or UL values of the 99% CI in the healthy volunteers. ^b^Low antioxidant levels: value below to LL with a 99% CI in the healthy volunteers. ^c^Readout TNF-*α* production, pg/ml. Low or high response of IFNGR was defined as TNF-*α* concentration outside LL or UL value of the 99% CI in the healthy volunteers. The IFNGR receptor increases <100% of the TNF-*α* concentration after LPS+rhIFN-*γ* vs. LPS alone. ^d^Readout IFN-*γ* production, pg/ml. Low or high response of IL-12R was defined as IFN-*γ* concentration outside LL or UL values of the 99% CI in the healthy volunteers. The IL-12 increases <100% of the IFN-*γ* production after rhIL-12 plus PHA stimulation with respect to PHA alone. CI: confidence interval; LL: lower limit; UL: upper limit; nd: not determined.

## Data Availability

Anonymous clinical data used to support the findings of this study are available from the corresponding author upon request.

## Supplementary Materials

Supplementary Materials Supplementary figure 1: graphical expression of the results of the immunological evaluation. As an aid in the interpretation of results, the graphical comparison of innate (a, b) and adaptive (c, d) responses between each patient and the mean of the control group are depicted.
