# Supplementary material for: Immunological Evaluation for Personalized Interventions in Children with Tuberculosis: Should It Be Routinely Performed?
Source: J Immunol Res. 2020 Sep 14;2020:8235149. doi: 10.1155/2020/8235149 (PMC7509549; doi:10.1155/2020/8235149)

Patient 2

Innate response

a NOD and TLR-dependent responses

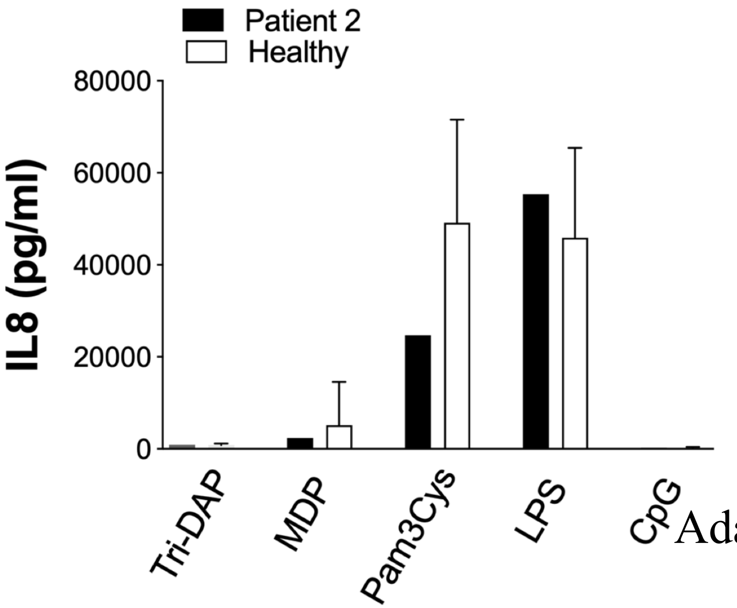

b Antioxidant levels

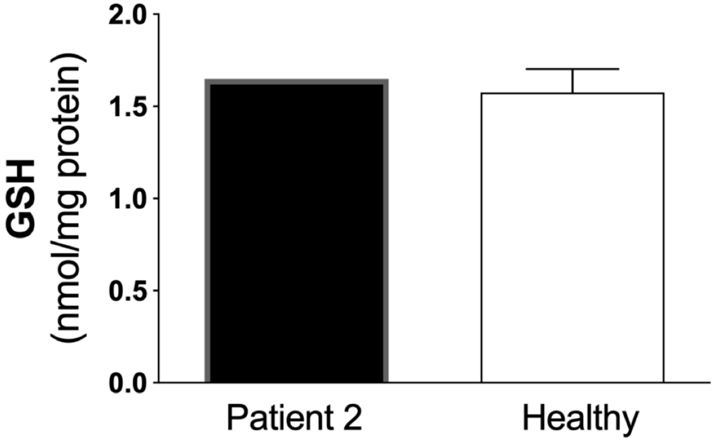

Adaptive response

c IL-12-dependent responses

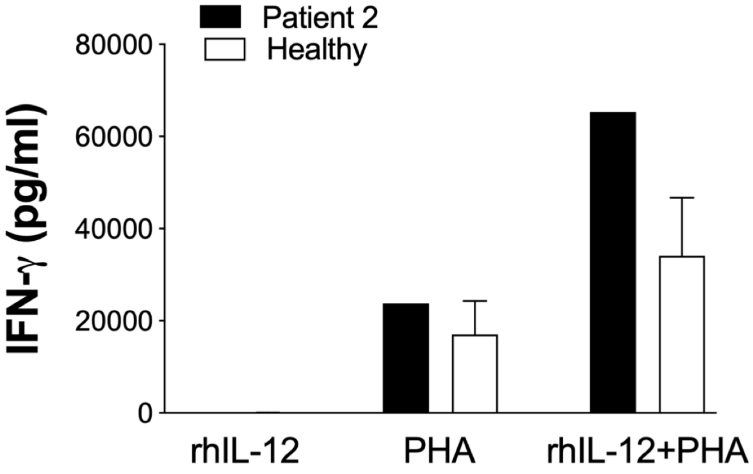

Patient 3

Innate response

a NOD and TLR-dependent responses

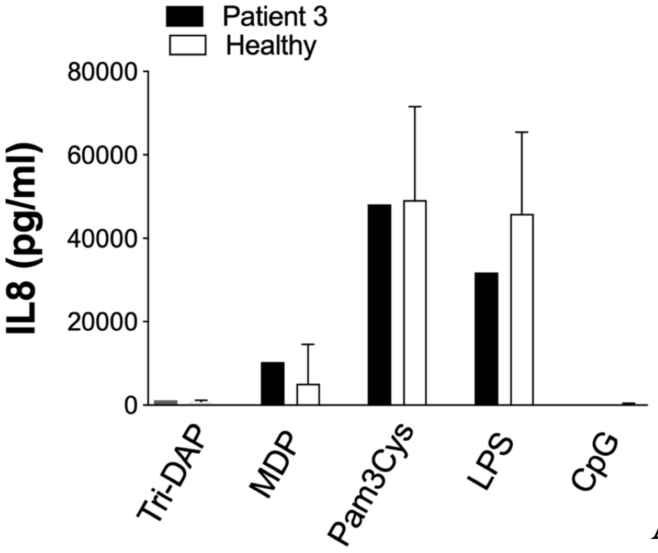

b Antioxidant levels

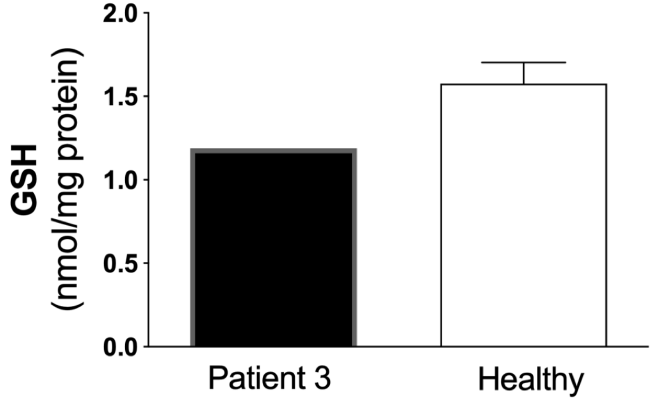

Adaptive response

c IFN-**γ**-dependent responses

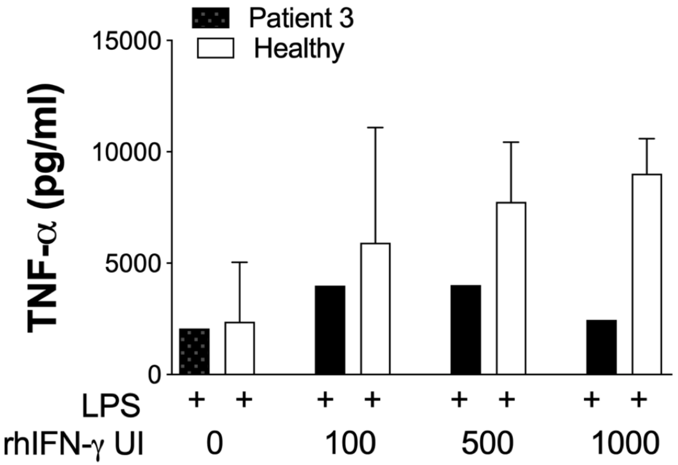

d IL-12-dependent responses

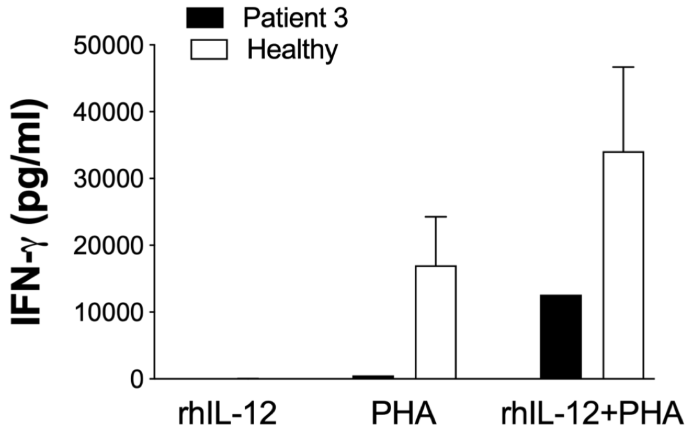

Patient 4

Innate response

a NOD and TLR-dependent responses

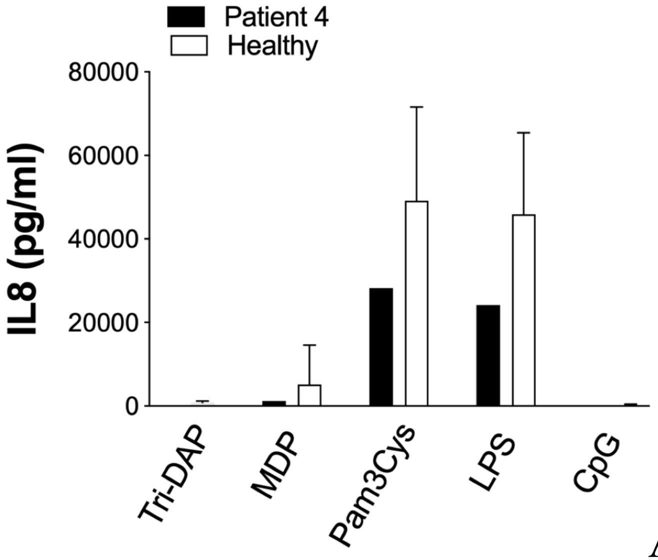

b Antioxidant levels

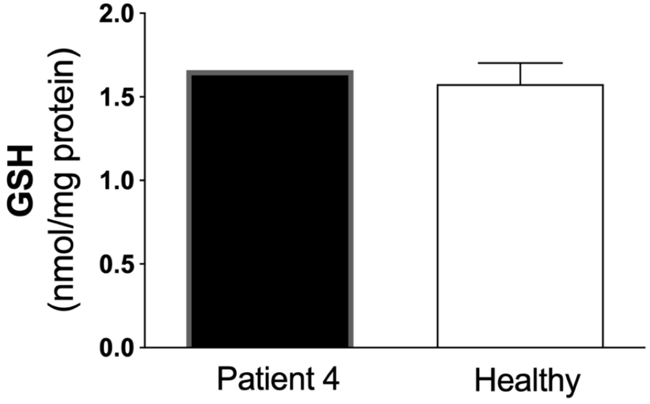

Adaptive response

c IFN-**γ**-dependent responses

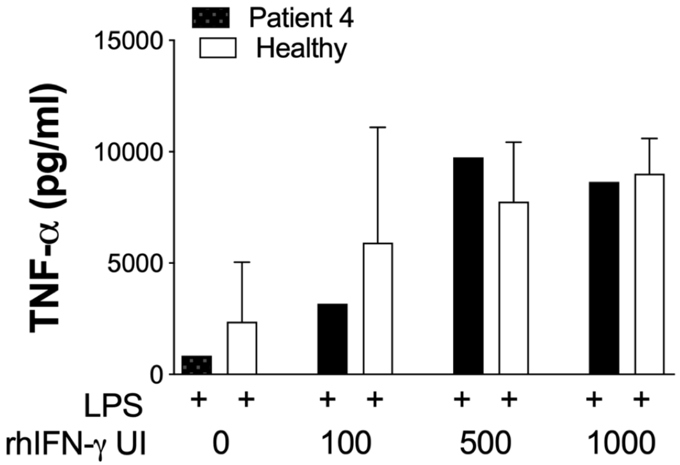

d IL-12-dependent responses

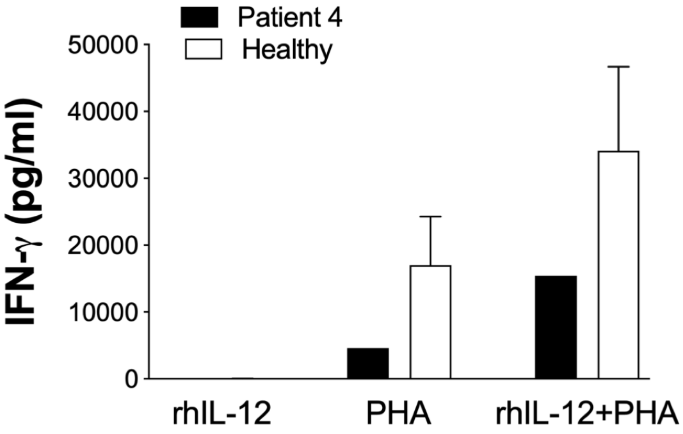

Patient 5

Innate response

a NOD and TLR-dependent responses

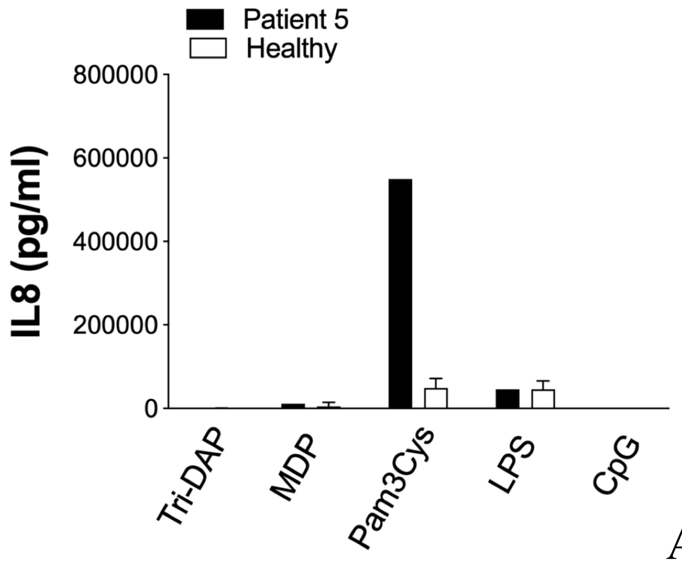

b Antioxidant levels

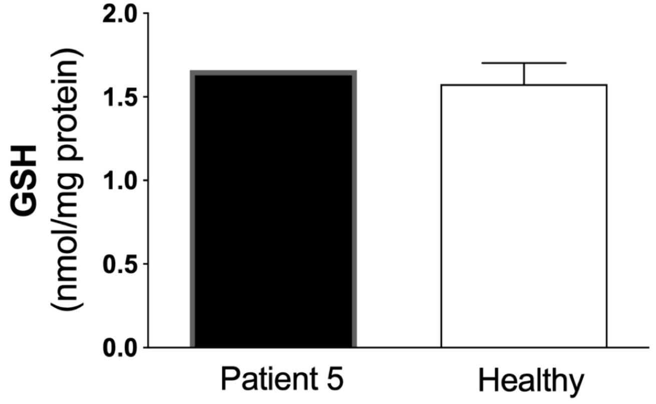

Adaptive response

c IFN-**γ**-dependent responses

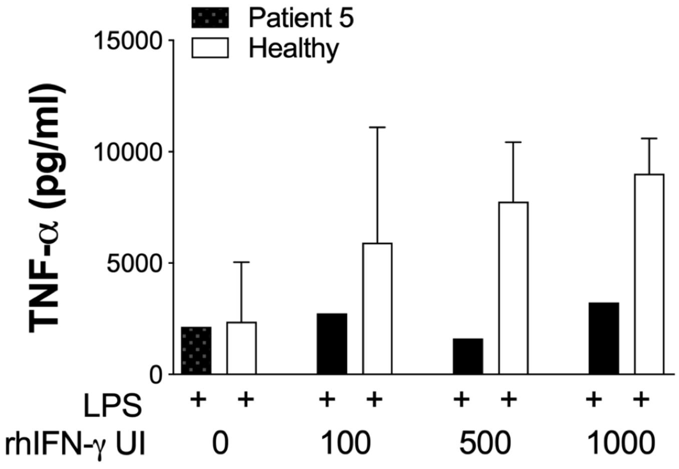

d IL-12-dependent responses

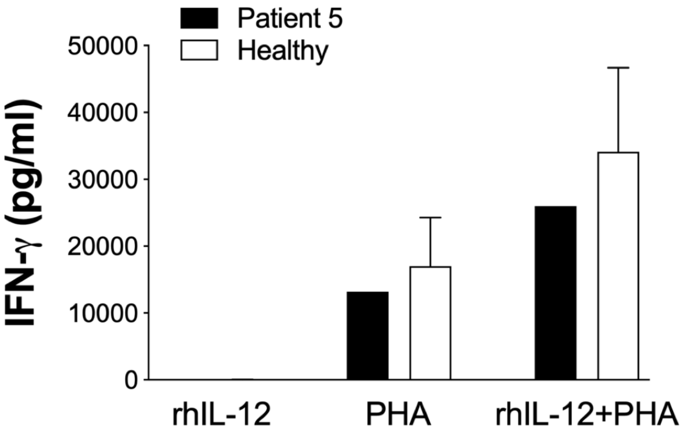

Patient 6

Innate response

a NOD and TLR-dependent responses

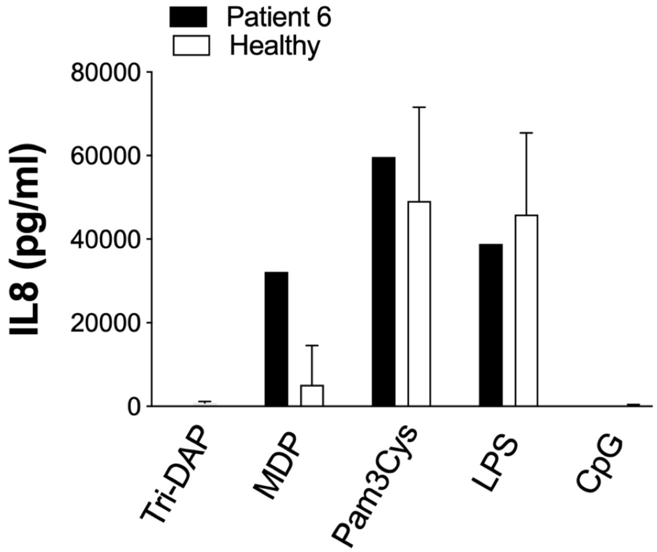

b Antioxidant levels

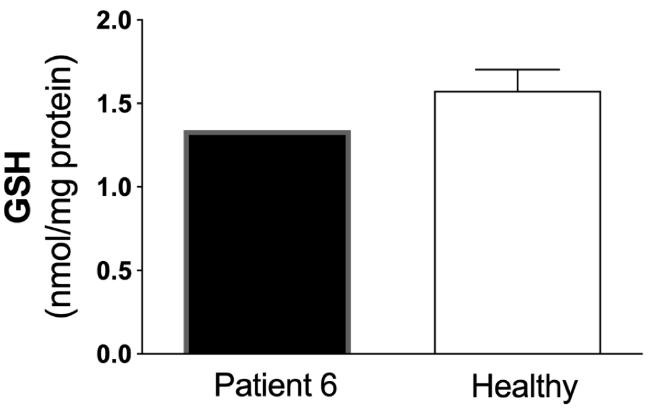

Adaptive response

c IFN-**γ**-dependent responses

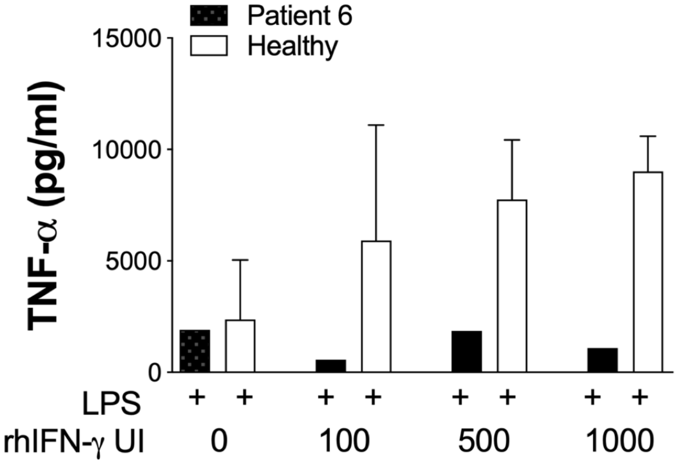

d IL-12-dependent responses

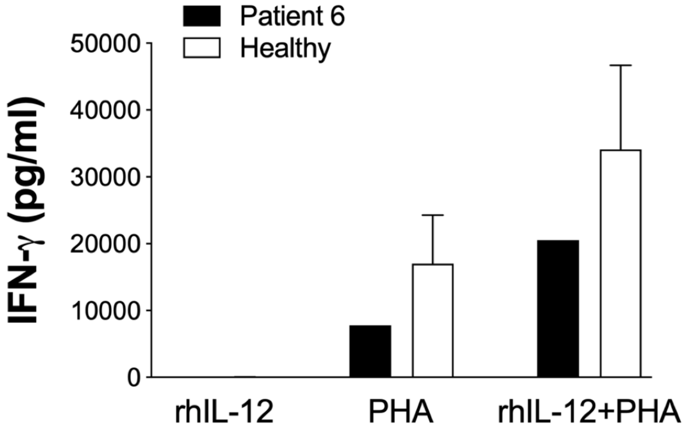

Patient 7

Innate response

a NOD and TLR-dependent responses

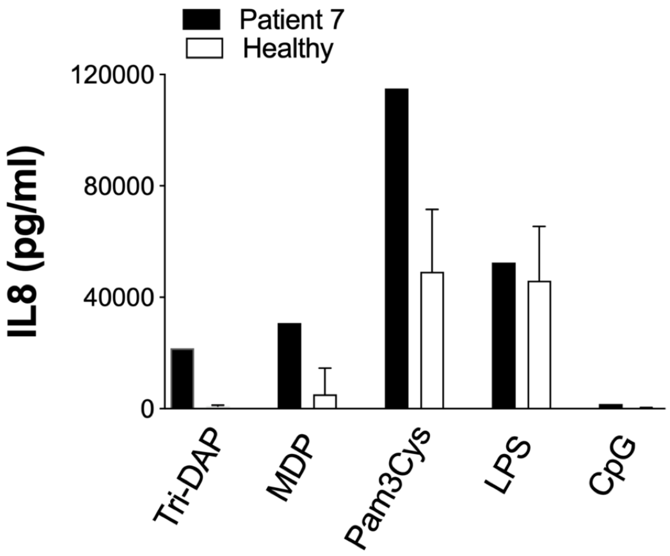

b Antioxidant levels

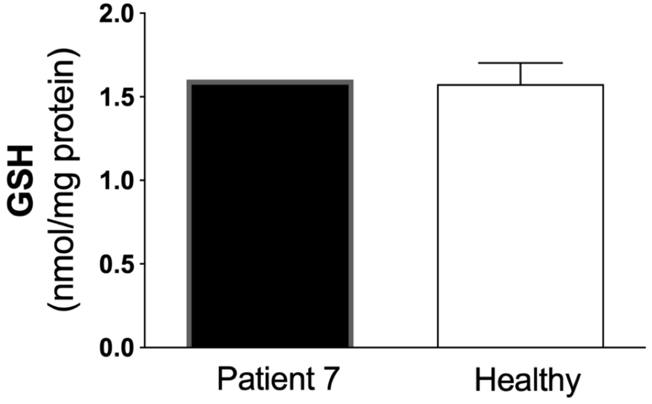

Adaptive response

c IFN-**γ**-dependent responses

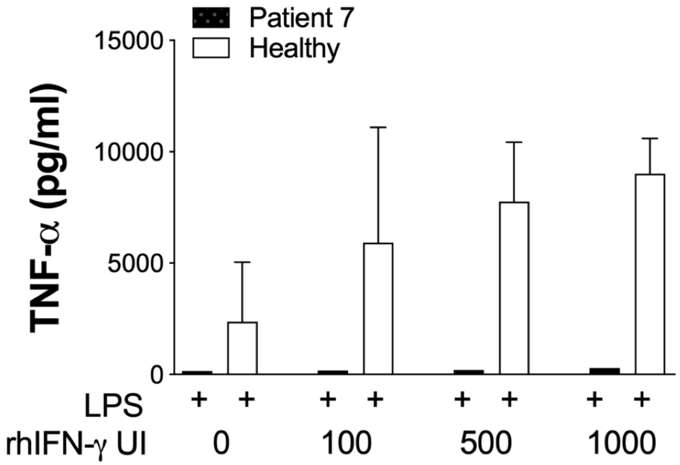

d IL-12-dependent responses

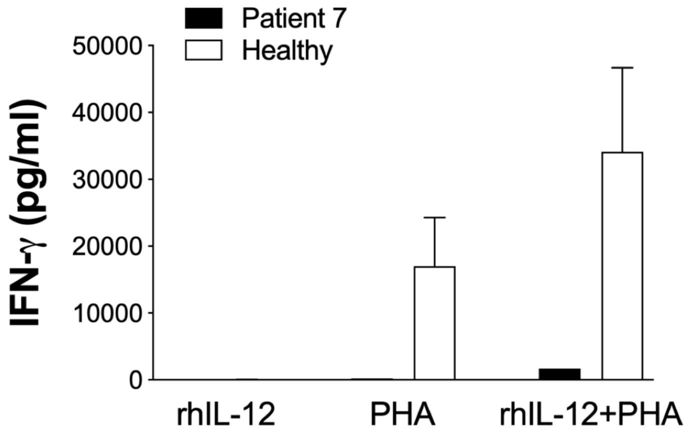

Patient 8

Innate response

a NOD and TLR-dependent responses

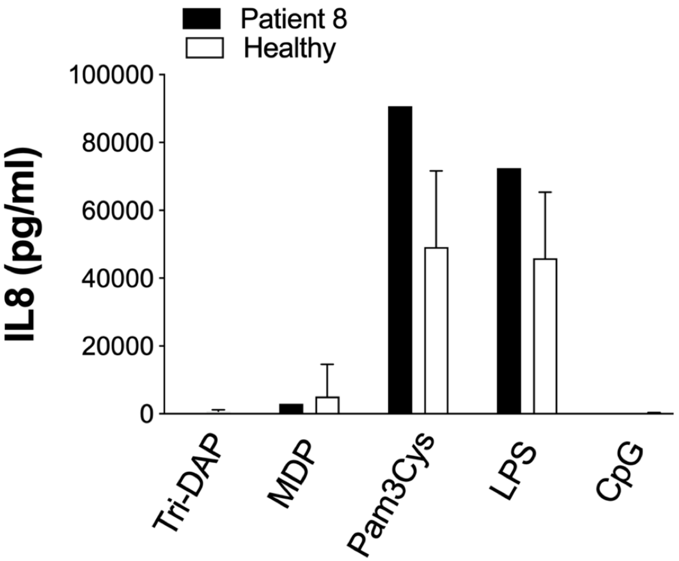

b Antioxidant levels

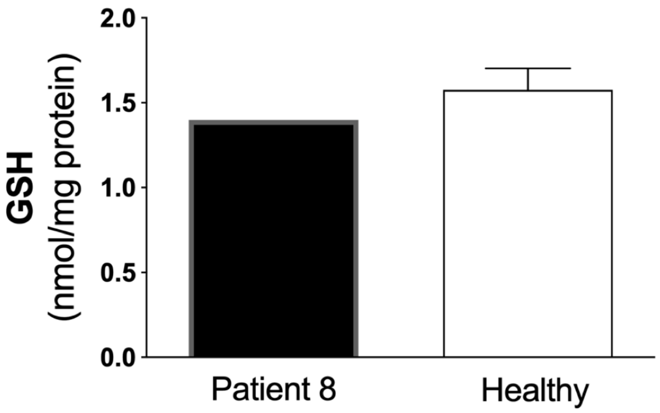

Adaptive response

c IFN-γ-dependent responses

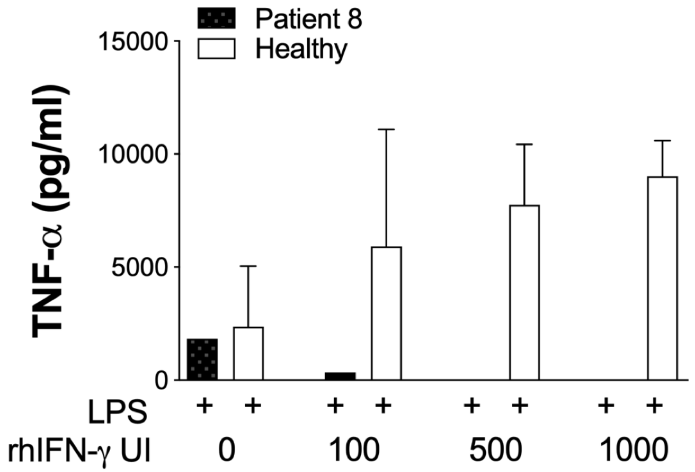

Supplement: Supplementary Materials — Supplementary figure 1: graphical expression of the results of the immunological evaluation. As an aid in the interpretation of results, the graphical comparison of innate (a, b) and adaptive (c, d) responses between each patient and the mean of the control group are depicted. [file 8235149.f1.pdf]
